# MD simulation studies to investigate iso-energetic conformational behaviour of modified nucleosides m^2^G and m^2^_2_G present in tRNA

**DOI:** 10.5936/csbj.201302015

**Published:** 2013-06-08

**Authors:** Rohit S Bavi, Susmit B Sambhare, Kailas D Sonawane

**Affiliations:** a Structural Bioinformatics Unit, Department of Biochemistry, Shivaji University, Kolhapur 416 004, Maharashtra, India; b Department of Microbiology, Shivaji University, Kolhapur 416 004, Maharashtra, India

## Abstract

Modified nucleic acid bases are most commonly found in tRNA. These may contain modifications from simple methylation to addition of bulky groups. Methylation of the four canonical nucleotide bases at a wide variety of positions is particularly prominent among the known modification. Methylation of N2 group of guanine is a relatively common modification in tRNA and rRNA. N2-methylguanosine (m^2^G) is the second most often encountered nucleoside in E. coli tRNAs. N2, N2- dimethylguanosine (m^2^_2_G) is found in the majority of eukaryotic tRNAs and involved in forming base pair interactions with adjacent bases. Hence, in order to understand the structural significance of these methylated nucleic acid bases we have carried out molecular dynamics simulation to see the salvation effect. The results obtained shows iso-energetic conformational behaviors for m^2^G and m^2^_2_G. The simulation trajectory of m^2^G shows regular periodical fluctuations suggesting that m^2^G is equally stable as either s-cis or s-trans rotamers. The two rotamers of m^2^G may interact canonically or non-canonically with opposite base as s-trans m^2^G26:C/A/U44 and s-cis m^2^G26:A/U44. The free rotations around the C-N bond could be the possible reason for these iso-energetic conformations. Dimethylation of G has almost no influence on base pairing with either A or U. Thus, these results reveal that modified nucleosides m^2^G and m^2^_2_G may play an important role to prevent tRNA from adopting the unusual mitochondrial like conformation.

## Introduction

RNA molecules undergo extensive post-transcriptional modifications that are important for their biological activities. Post-transcriptional modifications have been known as a natural mechanism to provide structural stability across the wide range of temperature in archaea as well as bacteria [1]. Transfer RNAs have the largest number and the greatest diversity of modifications: base or ribose methylation, base isomerization, base reduction, base thiolation and more complex hypermodifications [2, 3]. An important characteristic of tRNA is the presence of high content of modified nucleosides of which methylation represents the principle post-transcriptional modification during its maturation. In the maturation process of tRNA, transfer of methyl group occurs at polynucleotide level through an S-adenosyl-L-methionine donor, resulting in modification of heterocyclic base, the ribose moiety, or both [4]. The family of structurally related nucleosides m^2^G, m^2^_2_G, m^2^Gm and m^2^_2_Gm, are from known archaeal tRNA sequences. These modified nucleosides are conserved at only two locations, position 10 first base in the proximal position of the dihydrouridine (DHU) arm and at position 26, junction between the D-stem and the anticodon stem, where they play crucial roles in the control and stabilization of the tertiary L fold structure of the tRNA [5, 6]. The m^2^G and m^2^_2_G modifications in tRNA are found not only at position 26 but also at positions 6, 7, 9, 10, 18 and 27 in various organisms [7].

Experimentally, it has been found that level of certain modified nucleosides in archaeal thermophiles play major stabilizing role beyond the effects of magnesium ion binding and G-C content of tRNA [8]. Earlier study involving three-dimensional models of yeast tRNA^Phe^ derived from X-ray crystallographic data implies that m^2^_2_G26 functions as a molecular hinge.

This hinge adjusts the angular position of the D-stem and the anticodon stem during protein synthesis, thus maintaining a certain rigidity/flexibility in this part of tRNA [9]. Nuclear magnetic resonance studies on the resonance of the methyl proton in yeast tRNA^Phe^ also provide evidence to support the notation that m^2^_2_G26 has a significant role in regulating the stacking and conformational dynamics of this region of tRNA molecule [10]. The yeast tRNA (m^2^_2_G26) methyltransferase is dependent on the D-stem sequence and size of variable loop for the synthesis of N^2^-N^2^ dimethyl guanosine at 26^th^ position [11]. Mutations were introduced in both the D-stem and the variable loop of tRNA^Asp^ to obtain dimethylation of the normally unmodified G26 by the yeast N^2^, N^2^-dimethyl G26-methyltransferase [12]. The presence of m^2^_2_G26 in cytosolic tRNA may avert the molecule from adopting an unusual mitochondrial tRNA pattern folding and instead, allow it to fold into the canonical cloverleaf model. Through screening of the tRNA sequence and gene database it was revealed that some cytosolic tRNAs have the potential to fold into alternate structures. It was further noted that when a tRNA had the potential for this alternate folding, m^2^_2_G was found at position 10 and 26 presumably to block the formation of this non-standard folding pattern [13]. The methylated guanosine from 26 position of tRNA may have role in regulating the stacking interactions and the conformational dynamics [14].

N^2^-methylguanosine is found in both helical and loop regions of RNA secondary structure [15, 16] and it can exist in either *s-cis* or *s-trans* rotamers [18]. Incorporation of m^2^G was found to be iso-energetic with G in the duplex context as well as in GNRA (N = any nucleotide and R = purines) tetra loops [17]. The two rotamers of m^2^G, *s-cis* and *s-trans* have been found equally stable in RNA duplex [17] and in tRNA [18]. Free rotations around C-N bond aids m^2^G to get energetically two stable conformations and to form base pair interactions such as *s-cis* m^2^G:A/U or *s-trans* m^2^G:C/A/U [18]. This phenomenon has not been studied in detail at atomic level. Hence, present attempt has been made to investigate the dynamic behavior of iso-energetic conformations of m^2^G/m^2^_2_G using MD simulation technique. The results clearly show that the iso-energetic nature of *s-trans* and *s-cis* conformations could be because of free rotations around the C-N bond. Before this various computational techniques have been used to understand the conformational behavior and dynamics of many complex modified nucleosides [18–23]. However, all-atom molecular dynamics simulations of entire solvated ribosome, mRNA and tRNA complex have been studied to find out motion of tRNA from the A/T state into the A site [24].

**Figure 1:**
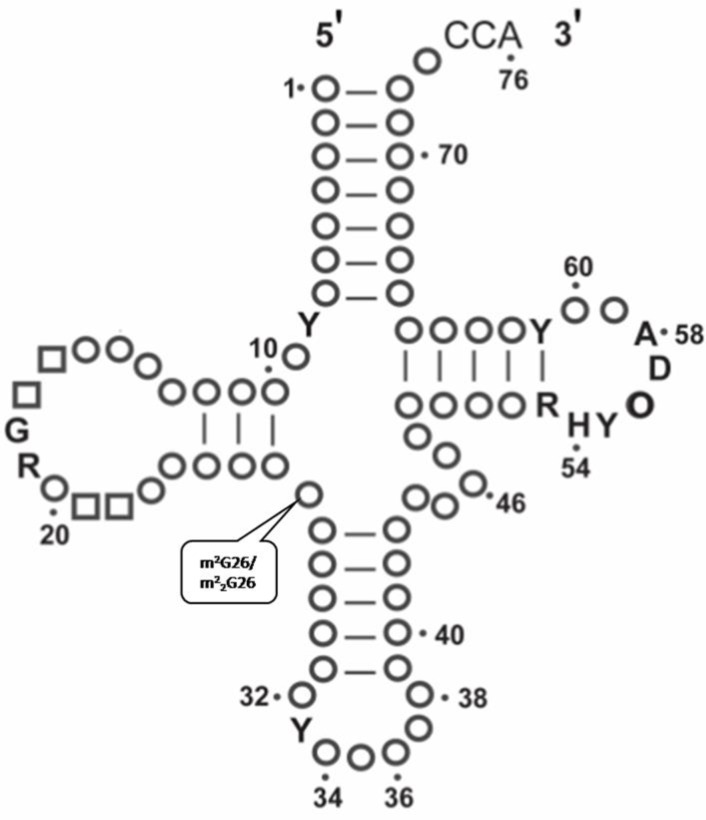
The cloverleaf structure showing m^2^G and m^2^_2_G at 26th position.

## Computational details

Molecular dynamics (MD) simulations were performed using Amber 10 simulation suite on (HP ProLiant-ML150G6) server in order to highlight the influence of explicit solvation on the conformation of modified nucleosides N^2^-methylguanosine (m^2^G) and N^2^-N^2^ dimethyl guanosine (m^2^_2_G). PCILO predicted preferred conformations of m^2^G and m^2^_2_G [18] were used as starting geometries for MD simulation studies. Antechamber software was used to calculate charges. Each nucleoside was solvated by 729 SPC/E water molecules filling a 34.20 × 31.05 × 31.85 Å^3^ rectilinear box with 1.0 water density [25]. Simulations were performed under periodic boundary conditions by employing the Particle Mesh Ewald [26] method to calculate long-range electrostatic interactions. MD trajectories were propagated at 2.0 fs time step using the shake algorithm [27] to all hydrogen atoms with non-bonded cutoff of 10 Å. The non-bonded pair list was updated by every 10 steps. The trajectories were calculated by maintaining constant temperature (300 K) and constant pressure (1atm) at 2 fs time step according to Berendsen coupling algorithm [28].

An equilibration protocol similar to the earlier molecular dynamics simulation study of nucleic acids was applied [29, 30]. The equilibration protocol consisted of 5000 steps of steepest descent minimization followed by 5 ps of MD at 300 K applied to relaxation of initial strain present between water molecules and N^2^-methyl derivatives of guanosine. In the next step N^2^-methyl derivatives were fixed while water molecules were allowed to relax at 100 K (1 ps), 200 K (1 ps), and finally at 300 K for 198 ps, thus equilibration protocol was completed at 200 ps.

Equilibrated system was further subjected to 5000 steps of steepest descent minimization to remove bad contacts between water molecules and nucleic acid bases. In further steps of MD simulation, no positional constrains were applied to the system and the temperature was progressively increased to 300 K in steps of 50 K with 1 ps at each step. Finally system was subjected to production MD of 20 ns at 300 K temperature and constant pressure (1atm) with fully solvated and neutralized system. PTRAJ module of Amber Tool 10 was used for analysis of average structures [31].

## Results and Discussion

### Dynamic behavior of N^2^-methylguanosine (m^2^G)

**Figure 2:**
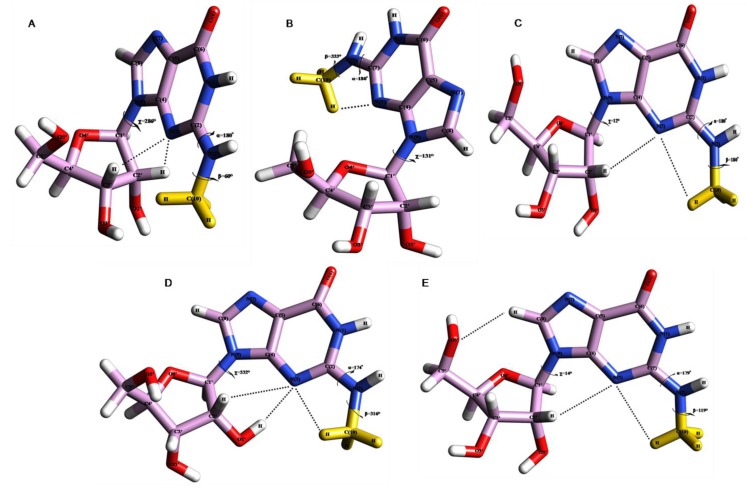
(**A**) PCILO predicted most stable structure of N2-methylguanosine (m^2^G) [18]. The methyl group has been given ‘Yellow’ colour for clear identification (**B**) Average structure of N2-methylguanosine for 0-1 ns. (**C**) Average structure of N2-methylguanosine for 3-4.5 ns. (**D**) Snapshot structure of N2-methylguanosine at 12 ns. (**E**) Snapshot structure of N2-methylguanosine at 20 ns.

**Figure 3:**
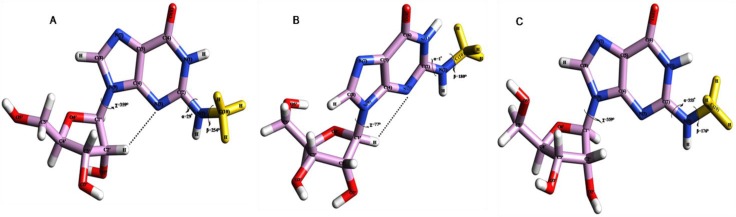
The methyl group has been given ‘Yellow’ colour for clear identification (**A**) Snapshot structure of N2-methylguanosine taken at 2 ns. (**B**) Average structure of N2-methylguanosine for 5-11ns. (**C**) Average structure of N2-methylguanosine for 13-19 ns.

**Figure 4:**
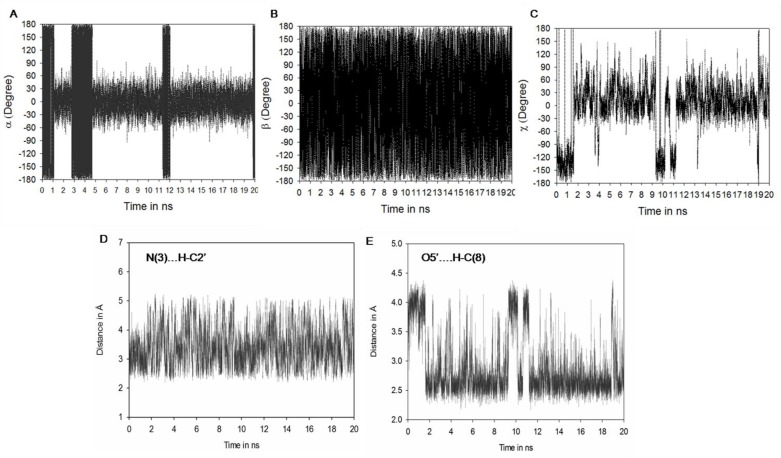
Molecular dynamics (MD) result: (**A**) Showing fluctuations in a torsion angle. (**B**) Fluctuations in torsion angle. (**C**) Fluctuations in χ torsion angle. (**D**) Fluctuations in hydrogen bonding between N(3)-HC2' (**E**) O5'-HC(8)

The preferred conformation of N^2^-methylguanosine [18] (Fig. 2A) has been used as a starting geometry for 20 ns molecular dynamics simulation study. In order to confirm the iso-energetic conformational behavior of m^2^G we have analyzed four different average structures particularly at 0 to 1 ns (Fig. 2B), 3 to 4.5 ns (Fig. 2C), 5 to 11 ns (Fig. 3B) and 13 to19 ns (Fig. 3C) and three snapshot structures particularly at 2ns (Fig. 3A), 12ns (Fig. 2D) and 20ns (Fig. 2E) of 20ns total simulation period. The geometrical parameters are mentioned in table 1. The selection of average and snapshot structures have been made based on the conformational flexibility observed during the MD simulation trajectory (Fig. 4,6) similarly as per our earlier conformational studies of yW [20], OHyW [21] and ac^4^C [32].

**Table 1:** Geometrical parameters for torsion angles and hydrogen bonding interactions for average and snapshot structures after MD simulation.

Modified nucleoside	Average structure at time (ns)	Torsion angle (degree)	Atoms involved (Atom 1 - Atom 2 - Atom 3)	Distance atom pair Atom1- Atom -2(Å)	Angle Atom 1- Atom 2- Atom-3 (degree)	Figure Ref.
m^2^G	PCILO most stable structure [18]	α = 180°, β = 60°, χ = 286°	N(3)…H-C2′	1.992	117.02	2A
			N(3)…H-C3′	2.269	113.78	
	0-1	α = 180°, β = 333°, χ = 131°	N(3)…H-C(10)	2.776	97.41	2B
	2	α = 29°, β = 254°, χ = 340°	N(3)…H-C2′	2.503	125.19	3A
	3-4.5	α = 180°, β = 180°, χ = 12°	N(3)…H-C2′	2.898	103.42	2C
			N(3)…H-C(10)	2.756	93.21	
	05-11	α = 1°, β = 180°, χ = 77°	N(3)…H-C1′	2.705	106.51	3B
	12	α = 174°, β = 316°, χ = 333°	N(3)…H-O2′	2.046	158.15	2D
			N(3)…H-C(10)	2.537	91.65	
			N(3)…H-C2′	2.902	93.55	
	13-19	α = 355°, β = 176°, χ = 359°	-	-	-	3C
	20	α = 179°, β = 119°, χ = 14°	O5′…H-C(8)	2.45	138.65	2E
			N(3)…H-C(10)	2.838	93.97	
			N(3)…H-C2′	2.947	102.34	

m^2^_2_G	PCILO most stable structure [18]	α = 0°, β = 60°, γ = 60°, χ = 286°	N(3)…H-C2′	1.992	117.02	5A
			N(3)…H-C3′	2.269	113.78	
	2-3	α = 348°, β = 178°, γ = 179°, χ = 6°	-	-	-	5B
	19-20	α = 99°, β = 178°, γ = 176°, χ = 359°	O5′…H-C(8)	1.587	106.52	5C

### Stabilization of s-trans m^2^G26 conformation

The MD simulation average structures for m^2^G taken at 0 to 1 ns (Fig. 2B), 3 to 4.5 ns (Fig. 2C), and snapshot structures at 12 ns (Fig. 2D) and 20 ns (Fig. 2E) shows the “proximal” or *s-trans* orientation with imidazole ring of guanosine.

The Methyl group of m^2^G point towards the N(3) atom of guanosine as observed in earlier study [18]. This *s-trans* or proximal orientation would allow Watson-Crick base pairing of m^2^G26 with C44 and non Watson-Crick base pairing with A/U44 at the hinge region of tRNA. Similar kind of *s-trans* orientation for m^2^G has been observed in our earlier conformational study [18] along with crystal conformer of m^2^G10 [33], where it forms Watson-Crick base pairing with C25.

The average structure obtained at 0 to 1 ns (Fig. 2B) maintains the initial geometry (Fig. 2A) [18] by preserving *s-trans* or “proximal” conformation for N^2^-methyl substituent of guanosine (26^th^), which is stabilized by hydrogen bonding interaction between N(3)…HC(10) (Fig. 2B and Table 1). This average structure (Fig. 2B Table 1) shows deviations for torsion angle β by 87° and χ by 155° whereas α retains its initial geometry [18] as found in crystal conformer 1EHZ.pdb [33] and 6TNA.pdb [9]. A large deviation around the torsion angle β is due to rotations around C-N bond. Next average structure taken at 3 to 4.5 ns (Fig. 2C) is also stabilized by hydrogen bonding between N(3)…HC(10) along with this, interaction between N(3)…HC2′ (Fig. 2C and Table 1) provides an additional structural stability to this average structure (Fig. 2C), as observed in our earlier conformational study of m^2^G [18]. The torsion angle β shows large deviation (120°) whereas α maintains starting value as compared with initial structure of m^2^G [18].

Snapshot structure selected at 12 ns (Fig. 2D) prefers *s-trans or “*proximal” conformation for m^2^G and stabilized by N(3)…HC(10), N(3)…HC2′ and N(3)…HO2′ interactions (Fig. 2D and Table 1) similar to earlier results of m^2^G [18]. This snapshot structure shows similar conformation for torsion angle α while torsion angles β and χ deviates to large extent from initial structure (Fig. 2A) as observed in crystal structure 1OB5.pdb [34].

Second snapshot structure (Fig. 2E and Table 1) selected at final trajectory (20 ns) of simulation study also preserves *s-trans* conformation for m^2^G and stabilized by intramolecular interactions between N(3)…HC(10), N(3)…HC2′ and O5′…HC(8) similar to the starting geometry (Fig. 2A) and PCILO preferred conformation of m^2^G obtained without glycosyl torsion angle rotation (χ = 16) [18]. Hence, this *s-trans* conformation of m^2^G would form canonical Watson-Crick base pairing interaction with C44 and non-canonical Watson-Crick base pairing with A/U44 in order to provide structural stability to the tRNA molecule during protein biosynthesis process similarly as observed in earlier conformational and sequence analysis studies [18].

### Stabilization of s-cis m^2^G26 conformation

The MD simulation snapshot structure selected at 2 ns (Fig. 3A), and average structures at 5 to 11 ns (Fig. 3B) and 13 to 19 ns (Fig. 3C) shows *s-cis* orientation for methyl substituent of m^2^G which point towards the N(1) atom of guanosine.

This orientation of N^2^-methylguanosine allows non Watson-Crick base pairing with adenosine (A44) and uracil (U44) instead of usual Watson-Crick base pairing with cytosine (C44) at the hinge region of tRNA. This *s-cis* conformational behavior of m^2^G was also noticed in our earlier study [18] and in tRNA^Phe^ crystal structure when m^2^G is present at 10th position [34]. The usual Watson-Crick base pairing between m^2^G10:C25 is not feasible when m^2^G prefers *s-cis* orientation as observed in crystal conformer (PDB ID: 1OB5) [34] instead it would form other non Watson-Crick base pairing interactions with A44 and U44 at the hinge region of tRNA.

The geometrical parameters for torsion angles and hydrogen bonding interactions analyzed from average and snapshot structures are given in table 1. The snapshot structure for m^2^G (Fig. 3A) taken at 2 ns prefers *s-cis* conformation due to change in α torsion angle which deviates from 180° to 29°, while other torsion angles β and χ diverges to great extent from initial structure and are in close agreement with crystal conformer 1OB5.pdb [34]. This structure is stabilized by the hydrogen bond between N(3)…HC2′ (Table 1) as found in earlier conformational study of m^2^G [18]. Average structure (Fig. 3B) chosen for the period 5 to 11 ns when α torsion angle flipped by 179° as compared to preferred structure of m^2^G (Fig. 2A).

The obtained average structure maintains distal conformation for methyl substituent of guanosine and gets stabilized by N(3)…HC1′ interaction which was not observed in *s-trans* conformer of m^2^G. Last average structure (Fig. 3C) was taken within the range of 13 to 19 ns, showing *s-cis* conformation of m^2^G. The *s-cis* conformation is obtained due to change in α torsion angle from 180° to 355°. This average structure (Fig. 3C) shows deviations for torsion angle β by 116° and χ by 73°. Obtained average structure (Fig. 3C) shows similar values for torsion angle α and χ as compared with crystal structure 1OB5.pdb [34]. A large deviation around the torsion angle β is due to fluctuations from *s-trans* to *s-cis* conformation by rotating C-N bond of methyl group.

### Fluctuations in torsion angles of N^2^-methylguanosine (m^2^G) during MD simulation

Analyses were also made for torsion angles and hydrogen bonding interactions of m^2^G during 20 ns simulation period (Fig. 4). The torsion angle α fluctuates periodically between *s-trans* (±180°) and *s-cis* (0°) rotamers of m^2^G during total simulation period (Fig. 4A and Table 1). For simulation time 0 to 1 ns, 2.8 to 4.7 ns, 11.5 to 12 ns and 20 ns (Fig. 4A) torsion angle α prefers *s-trans* orientation which is supported by weak interaction between N(3)…HC2′ (Fig. 4D) and N(3)…HC(10) (Table 1). Orientation of α torsion angle favors the usual Watson-Crick base pairing of m^2^G26 with C44 and unusual non-Watson-Crick base pairing with A/U44. Whereas, during simulation period 1 to 2.8 ns, 4.8 to 11.2 ns and 12 to 19.8 ns methyl substituent of guanosine prefers *s-cis* orientation which is stabilized by N(3)…HC2′ and N(3)…HC1′ hydrogen bonding interactions.

The *s-cis* conformation of N^2^-methyl substituent has also been observed in crystal structure when m^2^G present at 10th position in tRNA^Phe^ [34]. This orientation allows the non Watson-Crick base pairing between *s-cis* m^2^G with A/U44. Similar results were found in earlier conformational energy calculations performed over m^2^G [18].

Torsion angle β (Fig. 4B) maintains starting geometry ±180° [18] with small fluctuations at ± 60° as found in crystal structure 6TNA.pdb [9], 1EHZ.pdb [33] and 1OB5.pdb [34]. The glycosyl torsion angle (χ) (Fig. 4C) fluctuates at ±30°, ±120°, ±180° and favors the respective *anti* (1.8 to 9.2 ns, 11.2 to 18.9 ns and 19.2 to 20 ns) and *syn* (0 to 1.7 ns, 9.3 to 10.2, 10.8 to 11 ns and 19ns) conformation for m^2^G. The m^2^G is preferably stable at both *syn* and *anti* conformation, which allows usual (Watson-Crick) as well as unusual (non Watson-Crick) base pairing with C/A/U44. The syn conformation of *s-trans* and *s-cis* m^2^G is supported by O5′…HC(8) hydrogen bonding interaction whereas in *anti* conformation of *s-trans* and *s-cis* m^2^G, is held by N(3)…HC2′ and N(3)…HC(10) (Table 1) during MD simulation. Hydrogen bonding between O5′…HC(8) is varied in accordance with the fluctuations found in glycosyl torsion angle (χ) of m^2^G26 during simulation period.

### Molecular dynamics (MD) simulation study of N^2^-N^2^ dimethyl guanosine (m^2^_2_G)

**Figure 5:**
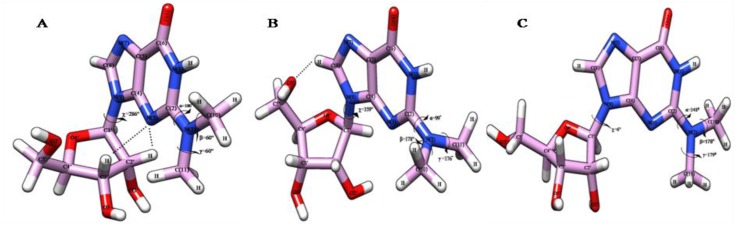
(**A**) PCILO predicted most stable structure of N2, N2-dimethylguanosine (m^2^_2_G) [18]. (**B**) Average structure of N2, N2-dimethylguanosine for 2-3 ns. (**C**) Average structure of N2, N2-dimethylguanosine for 19-20 ns.

In order to see solvation effect on N^2^-N^2^ dimethylguanosine explicit molecular dynamics simulation study of 20 ns has been performed over the PCILO preferred conformation (Fig. 5A) [18]. To confirm the conformational behavior of m^2^_2_G we have analyzed two different average structures taken at 2-3 ns (Fig. 5B) and last 1000 ps from 19-20 ns (Fig. 5C), their geometrical parameters are listed in table 1.

**Figure 6:**
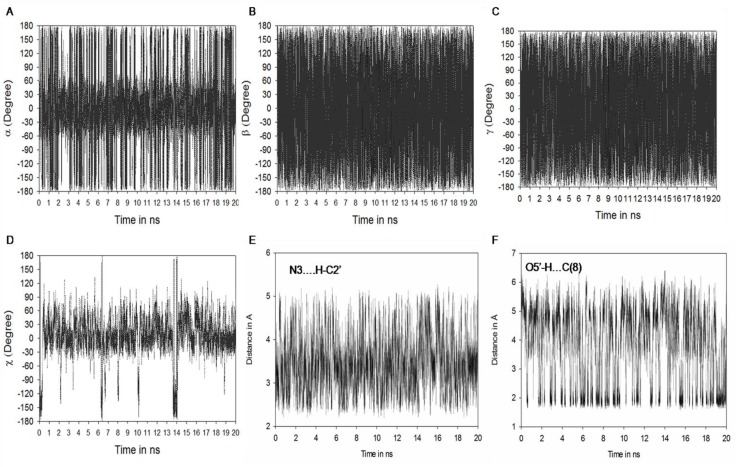
Molecular dynamics (MD) result: (**A**) Showing fluctuations in a torsion angle. (**B**) Fluctuations in torsion angle. (**C**) Fluctuations in γ torsion angle. (**D**) Fluctuations in χ torsion angle. (**E**) Fluctuation in hydrogen bonding between N(3)-HC2' (**F**) O5'-HC(8)

The average structure obtained from 2 to 3 ns (Fig. 5B) prefers distal conformation for m^2^_2_G and prevent Watson-Crick base pairing with C, instead it would prefer non-canonical Watson-Crick interactions to pair with A/U44. Compared with crystal conformer (1EHZ.pdb) [33], average structure retains quite similar torsion angle values for α, β and χ. The average structure selected at last 1000 ps (19 to 20 ns) does not show much difference as compared with earlier average structure. The only difference between these two average structures is variation around α torsion angle which is positioned to 99° (Fig. 5C) from its preferred value (Fig. 5A). Due to this small change in conformational property of m^2^_2_G, dimethylation of guanosine has almost no influence on pairing with either A or U, because the N^2^ position of guanosine has no impact on these base pairing interactions. The m^2^_2_G-A pair can be formed with little hindrance, because, even though the methyl groups of m^2^_2_G26 are in the plane of the base, as in case of yeast tRNA^Phe^_,_ they take part in a propeller-type arrangement with the base. The m^2^_2_G-U pair would not be affected by any conformational arrangement of the methyl groups similarly as discussed in [18].

### Fluctuations in torsion angles of N^2^, N^2^- dimethylguanosine (m^2^_2_G) during MD simulation

The α torsion angle fluctuates periodically in between ±180° or ± 60° (Fig. 6A and Table 1) over the 20 ns molecular dynamics simulation period suggesting free rotation around C-N bond.

Torsion angles β (Fig. 6B) and γ (Fig. 6C) retain preferred values ±180°, with small fluctuations at ±60° throughout the simulation period. Glycosyl torsion angle (χ) adopts *anti* conformation during the simulation study. Such type of anti conformation for N^2^,N^2^-dimethylguanosine was confirmed through crystal structure (1EHZ.pdb, 1EVV.pdb, 1OB5.pdb 6TNA.pdb).

## Conclusion

The regular periodical fluctuations around the bond C(2)-N(2) of m^2^G was observed throughout the 20 ns molecular dynamics simulation, which confirms the existence of iso-energetic *s-cis* or *s-trans* rotamers of m^2^G. These iso-energetic rotamers interconvert easily during the simulation period. These results are in favor with preferred and alternative conformations of m^2^G obtained by our earlier conformational energy calculations [18] as well as crystal structure (1EHZ.pdb [33] and 1OB5.pdb [34]). The periodical fluctuations of *s-trans* to *s-cis* and vice versa could be possible due to free rotations around the C-N bond of methyl group. According to tRNA sequence analysis [18] and this MD simulation results we would like to say that m^2^G26 can form three different canonical as well as non-canonical Watson-Crick base pairing interactions with other bases. Such base pairing may be summarized as i) an usual Watson-Crick base pairing of m^2^G26-C44 where the methyl substituent must be in *s-trans* orientation, ii) non Watson-Crick base pairing between m^2^G26-A where the methyl substituent is likely to be *s-cis* orientation, and iii) non Watson-Crick m^2^G26-U base pairing where the methyl group can adopt one of them, i.e. *s-cis* or *s-trans* conformation. These results reveal that m^2^G is equally stable as either the *s-cis* or *s-trans* rotamers and the rotational preference of methyl group may be specific to the sequence context reliant upon which face of the base contributes in hydrogen bonding. Thus, MD simulation results confirm that the N^2^-methyl group of m^2^G26 may prefer energetically two stable rotamers, i.e., *s-trans* m^2^G26:C/A/U44 and *s-cis* m^2^G26:A/U44 as found in earlier results [18].

Similarly, the presence of two methyl groups unlike in case of single methyl in m^2^G virtually eliminates the possibility of pairing with C and, indeed, m^2^_2_G26 pairs exclusively with A or U at position 44 and is flanked by C27:G43 on one side and the m^2^G10-C25-G45 triple on the other [35]. Hence, these results suggest that the modified nucleosides m^2^G26 and m^2^_2_G26 play an important role in tRNA folding and may prevent tRNA from adopting the unusual mitochondrial like conformation.

## References

[1] Noon KR, Guymon R, Crain PF, McCloskey JA, Thomm M, et al (2003) Influence of temperature on tRNA modification in archaea: Methanococcoides burtonii (optimum growth temperature [Topt], 23 °C) and Stetteria hydrogenophila (Topt, 95 °C). Journal of Bacteriology 185: 5483–5490 12949100 10.1128/JB.185.18.5483-5490.2003 PMC193749

[2] Limbach PA, Crain PF, McCloskey JA (1994) Summary: the modified nucleosides of RNA. Nucleic Acids Research 22: 2183–2196 7518580 10.1093/nar/22.12.2183 PMC523672

[3] Limbach PA, Crain PF, Pomerantz SC, McCloskey JA (1995) Structures of posttranscriptionally modified nucleosides from RNA. Biochimie 77: 135–138 7541251 10.1016/0300-9084(96)88116-8

[4] Agris PF, Koh H, Soll D (1973) The effect of growth temperatures on the in vivo ribose methylation of Bacillus stearothermophilus transfer RNA. Archives of Biochemistry and Biophysics 154: 277–282 4689778 10.1016/0003-9861(73)90058-1

[5] Sprinzl M, Horn C, Brown M, Ioudovitch A, Steinberg S (1998) Compilation of tRNA sequences and sequences of tRNA genes. Nucleic Acids Research 26: 148–153 9399820 10.1093/nar/26.1.148 PMC147216

[6] Saenger W (1984) Principles of nucleic acid structure Springer-Verlag, New York, pp. 334–337

[7] Auffinger P, Westhof E (1998) Modification and Editing of RNA In Grosjean H, Benne R, editors, ASM Press, Washington DC pp. 569–576

[8] Kowalak JA, Dalluge JJ, McCloskey JA, Stetter KO (1994) The role of posttranscriptional modification in stabilization of transfer RNA from hyperthermophiles. Biochemistry 33: 7869–7876 7516708 10.1021/bi00191a014

[9] Sussman JL, Holbrook SR, Warrant RW, Church GM, Kim SH (l978) Crystal structure of yeast phenylalanine Transfer RNA 1. Crystallographic refinement. Journal of Molecular Biology 123: 607–630 357742 10.1016/0022-2836(78)90209-7

[10] Boyle J, Robillard GT, Kim SH (1980) Sequential folding of Transfer RNA: a nuclear magnetic resonance study of successively longer tRNA fragments with a common 5’ end. Journal of Molecular Biology 139: 601–625 6997498 10.1016/0022-2836(80)90051-0

[11] Edqvist J, Straby KB, Grosjean H (1995) Enzymatic formation of N2, N2 dimethylguanosine in eukaryotic tRNA: importance of the tRNA architecture. Biochimie 77: 54–61 7599276 10.1016/0300-9084(96)88104-1

[12] Edqvist J, Blomqvist K, Straby KB (1994) Structural elements in yeast tRNAs required for homologous modification of guanosine-26 into dimethylguanosine-26 by the yeast Trm1 tRNA-modifying enzyme. Biochemistry 33: 9546–9551 8068629 10.1021/bi00198a021

[13] Steinberg S, Cedergren R (1995) A correlation between N2-dimethylguanosine presence and alternate tRNA conformers. RNA 1: 886–891 8548653 PMC1369337

[14] Ginell SL, Parthasarathy R (1978) Conformation of N2-methylguanosine, a modified nucleoside of tRNA. Biochemical and Biophysical Research Communications 84: 886–894 728157 10.1016/0006-291x(78)91666-2

[15] Gutell RR, Gray MW, Schnare MN (1993) A compilation of large subunit (23S and 23S-like) ribosomal RNA structures. Nucleic Acids Research 21: 3055–3074 8332527 10.1093/nar/21.13.3055 PMC309733

[16] Gutell RR (1993) Collection of small subunit (16S- and 16S-like) ribosomal RNA structures. Nucleic Acids Research 21: 3051–3054 8332526 10.1093/nar/21.13.3051 PMC309732

[17] Rife JP, Cheng CS, Moore PB, Strobel SA (1998) N2-methylguanosine is iso-energetic with guanosine in RNA duplexes and GNRA tetraloops. Nucleic Acids Research 26: 3640–3644 9685477 10.1093/nar/26.16.3640 PMC147776

[18] Bavi RS, Kamble AD, Kumbhar NM, Kumbhar BV, Sonawane KD (2011) Conformational preferences of modified nucleoside N2-methylguanosine (m2G) and its derivative N2, N2-dimethylguanosine (m22G) occur at 26th position (hinge region) in tRNA. Cell Biochemistry and Biophysics 61: 507–521 21735129 10.1007/s12013-011-9233-1

[19] Sonawane KD, Sonawane UB, Tewari R (2002) Conformational preferences of anticodon 3’-adjacent hypermodified nucleic acid base cis- or trans-zeatin and its 2-methylthio derivatives cis- or trans-ms2zeatin. Journal of Biomolecular Structure and Dynamics 19: 637–648 11843625 10.1080/07391102.2002.10506770

[20] Kumbhar NM, Sonawane KD (2011) Iso-energetic multiple conformations of hypermodified nucleic acid base wybutine (yW) which occur at 37th position in anticodon loop of tRNAPhe. Journal of Molecular Graphics and Modelling 29: 935–946 21530341 10.1016/j.jmgm.2011.03.005

[21] Kumbhar NM, Kumbhar BV, Sonawane KD (2012) Structural significance of hypermodified nucleic acid base hydroxywybutine (OHyW) which occur at 37th position in the anticodon loop of tRNAPhe. Journal of Molecular Graphics and Modelling 38: 174–185 23073221 10.1016/j.jmgm.2012.07.005

[22] Sonawane KD, Tewari R (2008) Conformational preferences of hypermodified nucleoside lysidine (k2C) occurring at “wobble” position in anticodon loop of tRNAIle, Nucleosides. Nucleotides and Nucleic Acids 27: 1158–1174 10.1080/15257770802341475 18788046

[23] Sonawane KD, Sonavane UB, Tewari R (2000) Conformational flipping of the N(6) substituent in diprotonated N6-(N-glycylcarbonyl)adenines: The role of N(6)H in purine- ring- protonated ureido adenines. International Journal of Quantum Chemistry 78: 398–405

[24] Caulfield T, Devkota B (2012) Motion of transfer RNA from the A/T state into the A-site using docking and simulations. Proteins 80: 2489–2500 22730134 10.1002/prot.24131

[25] Berendsen HJC, Griegera GR, Straatsma TP (1987) The missing term in effective pair potentials. The Journal of Physical Chemistry 91: 6269–6271

[26] Darden T, York D, Pedersen L (1993) Particle Mesh Ewald: an N.log(N) method for Ewald sums in large systems. Journal of Chemical Physics 98: 10089–10092

[27] Ryckaert JP, Ciccotti G, Berendsen HJC (1977) Numerical integration of the cartesian equations of motion of a system with constraints: molecular dynamics of n-alkanes. Journal of Computational Physics 23: 327–336

[28] Berendsen HJC, Postma JPM, Van Gunsteren WF, DiNola A (1984) Molecular dynamics with coupling to an external bath. Journal of Chemical Physics 81: 3684–3690

[29] Auffinger PS, Loise-May S, Westhof E (1995) Multiple molecular dynamics simulation of the anticodon loop of tRNAAsp in aqueous solution with counter ions. Journal of the American Chemical Society 117: 6720–6726

[30] Auffinger PS, Loise-May S, Westhof E (1996) Hydration of C-H groups in tRNA. Faraday Discussions 103: 151–174 9136637 10.1039/fd9960300151

[31] Case DA, Darden TA, Cheatham TE III , Simmerling CL, Wang J, et al (2008) AMBER 10, University of California, San Francisco

[32] Kumbhar BV, Kamble AD, Sonawane KD (2013) Conformational Preferences of Modified Nucleoside N(4)-Acetylcytidine, ac^4^C Occur at ‘‘Wobble’’ 34th Position in the Anticodon Loop of tRNA. Cell Biochemistry and Biophysics DOI 10.1007/s12013-013-9525-8 23408308

[33] Shi H, Moore PB (2000) The crystal structure of yeast phenylalanine tRNA at 1.93 Å resolution: A classic structure revisited. RNA 6: 1091–1105 10943889 10.1017/s1355838200000364 PMC1369984

[34] Parmeggiani A, Krab IM, Watanabe T, Nielsen RC, Dahlberg C, et al (2006) Enacyloxin IIa pinpoints a binding pocket of elongation factor Tu for development of novel antibiotics. Journal of Biological Chemistry 281: 2893–2900 16257965 10.1074/jbc.M505951200

[35] Pallan PS, Kreutz C, Bosio S (2008) Effects of N2, N2 -dimethylguanosine on RNA structure and stability: Crystal structure of an RNA duplex with tandem m22G:A pairs. RNA 14: 2125–2135 18772248 10.1261/rna.1078508 PMC2553729

